# Long-Gap Esophageal Atresia Repair Using Staged Thoracoscopic Internal Traction: The First Kazakhstan Experience

**DOI:** 10.1089/lap.2022.0244

**Published:** 2022-12-08

**Authors:** Zhenis Sakuov, Damir Dzhenalaev, Marat Ospanov, Dastan Rustemov, Vasiliy Lozovoy, Asylzhan Erekeshov, Tolegen Otegen, Dariusz Patkowski

**Affiliations:** ^1^ Department of Pediatric Surgery, “National Scientific Center for Maternal and Child Health,” University Medical Center, Nur-Sultan, Kazakhstan.; ^2^ NJSC “Astana Medical University,” Nur-Sultan, Kazakhstan.; ^3^ Department of Pediatric Surgery and Urology, Wroclaw University of Medicine, Wroclaw, Poland.

**Keywords:** long-gap esophageal atresia, internal traction, thoracoscopic repair, newborn

## Abstract

**Introduction:**

The treatment of long-gap esophageal atresia (LGEA) remains an important issue for pediatric surgeons. There are several methods of treating LGEA with various advantages and disadvantages. Thoracoscopic esophageal elongation using internal traction sutures has been developed more recently. Therefore, we wanted to report on our first experience in treating such pathology using staged thoracoscopic internal traction.

**Objective:**

To share our first experience in the treatment of LGEA using staged thoracoscopic internal traction.

**Methods:**

Three children with LGEA were treated at the University Medical Center “National Scientific Center for Maternal and Child Health” in the Pediatric Surgery Department, Nur-Sultan, Kazakhstan, using the method of staged thoracoscopic internal traction.

**Results:**

At the age of 3–4 months, 3 patients were operated on successfully using staged thoracoscopic internal traction. In any case, converting to an open thoracotomy was not needed and no anastomotic leakage was observed. In 2 cases, stenosis occurred that was treated by dilatation at least twice, 1 child had no stenosis.

**Conclusions:**

Thoracoscopic internal traction technique for LGEA was performed for the first time in Kazakhstan that showed its safety and possible future use in the surgical treatment of this congenital malformation.

## Introduction

Long-gap esophageal atresia (LGEA) remains a significant concern for pediatric surgeons. To date, there is no unified treatment concept for such patients. Preservation of the native esophagus is believed to be the primary goal of surgical correction of esophageal atresia. Replacing the esophagus with the colon, jejunum, or stomach is technically difficult and has a high mortality rate.

Today, there are various ways to close the gap between the proximal and distal parts of the atretic esophagus, such as extrathoracic (thoracotomy, thoracoscopic) and intrathoracic traction methods to lengthen the esophagus.^1–4^ The purpose of this study is to present our first experience in the treatment of LGEA using staged thoracoscopic internal traction.

## Materials and Methods

From 2017 to 2021 at the University Medical Center “National Scientific Center for Maternal and Child Health” in the Pediatric Surgery Department, Nur-Sultan, Kazakhstan, 27 newborns were treated with esophageal atresia: type A—4 patients, type B—3 patients, type C—19 patients, and type D—1 patient. During this period, we successfully performed 15 trachea–esophageal repairs. In children with types A and B, as well as in children with type C in whom the primary esophageal anastomosis was unsuccessful, a gastrostomy and esophagostomy were placed as the first stage of surgical correction, followed by the replacement of the esophagus with the stomach or colon.

By 2021, 3 patients in whom esophageal atresia was detected antenatally (based on nonvisualization of the stomach and polyhydramnios) were born in our center. After birth, the diagnosis of LGEA was made by an X-ray contrast study. A gastrostomy tube was placed as the first step, and a tube was put into the upper pouch of the esophagus to prevent saliva aspiration and the patients were discharged for outpatient treatment. At the age of 3–4 months, children were readmitted to the hospital for the next step of surgical correction. During 7 days master class with the live participation of Professor D. Patkowski, staged thoracoscopic elongation using internal traction sutures was first performed in Kazakhstan.

**FIG. 1.**
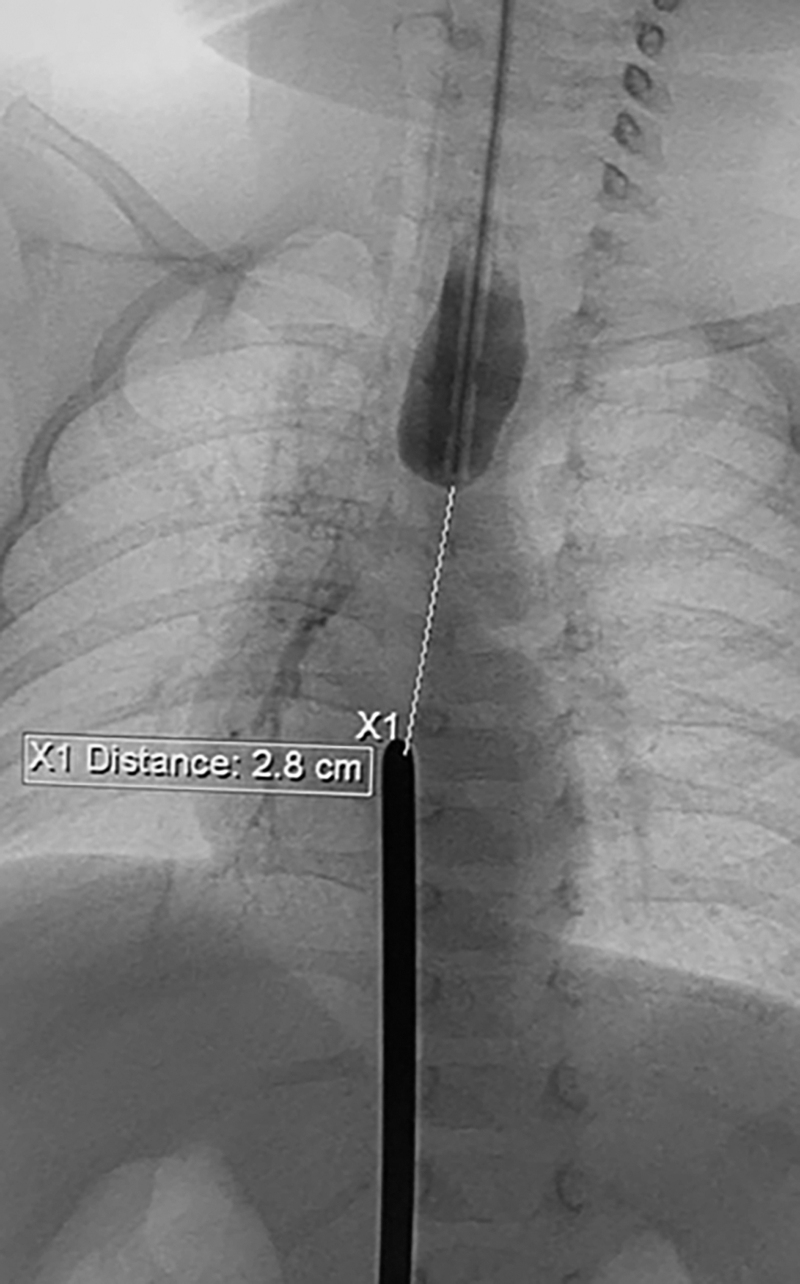
Measurement between esophageal ends.

By introducing radiopaque bougies, the diastasis between the proximal and distal pouches of the esophagus was measured (Fig. 1). The surgery began with rigid tracheoscopy to confirm and establish the level of the upper tracheoesophageal fistula: type A was detected in 1 child and type B was diagnosed in other 2 children, according to Gross classification (Table 1). Then, the children were placed in a prone position, with the right side of their bodies slightly elevated. In the VI intercostal space along the right posterior axillary line, 1.5 cm below the angle of the scapula, a 5 mm camera was installed in an open way.

**Table 1.** Patients' Demographic Data

Patient	No. 1	No. 2	No. 3
Gender	Female	Female	Male
Gross classification (type)	A	B	B
Birthweight (g)	3200	2547	2090
Gestational age (week)	39	41	34
Weight at correction (g)	7200	4170	3900

Under the control of a telescope, carbon dioxide was insufflated at a pressure of 6 mmHg with a flow rate of 1 L/min. Under camera control, two 3 mm ports were placed around the scapula. Dissection of the distal esophageal segment down to the diaphragm was performed without using electrocoagulation. The proximal esophageal segment was also mobilized to the cervical section; the upper tracheoesophageal fistula was ligated in 2 children using 4-0 absorbable sutures. After complete mobilization of esophageal segments, direct esophageal anastomosis is not possible. The ends of the esophagus were brought together by internal traction nonabsorbable sutures 2-0.

**FIG. 2.**
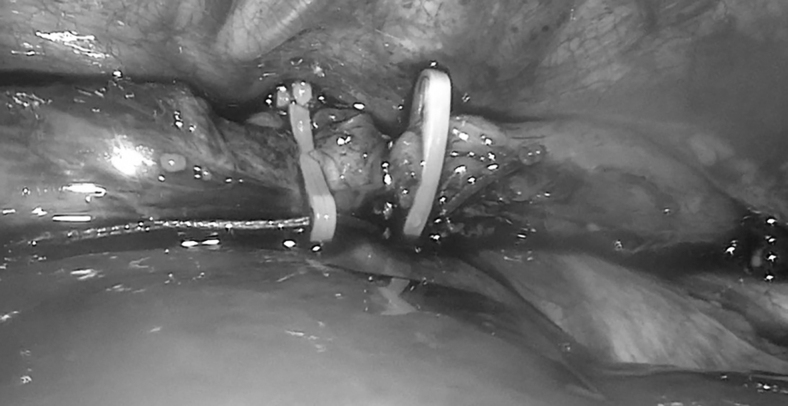
Thoracoscopic elongation of the esophagus.

Traction was carried out as far as possible using sliding nodes, which were reinforced with hem-o-lock clips (Fig. 2). To apply esophago-esophagoanastomosis, 2 patients needed one traction procedure and 1 infant needed two procedures. Then, the patients were transferred to the pediatric intensive care unit and the children were under full myorelaxation and sedation. The next stage of surgical correction was performed on average 2–4 days after traction (Table 2). After separation of pleuropulmonary adhesions, the proximal and distal ends of the esophagus were joined together without diastasis.

**Table 2.** Results of the Elongation of Esophagus Using Thoracoscopic Internal Traction Method

Patient	No. 1	No. 2	No. 3
Age, days	140	84	64
Gastrostomy	+	+	+
No. of tractions	1	2	1
Duration of traction, days	2	4/2^a^	3
Anastomosis leakage	—	—	—
Stenosis	+	—	+
Gastroesophageal reflux	+	+	+
Feeding	Complete mouth feeding	Complete mouth feeding	Complete mouth feeding

^a^ First traction was 4 days, second was 2 days.

**FIG. 3.**
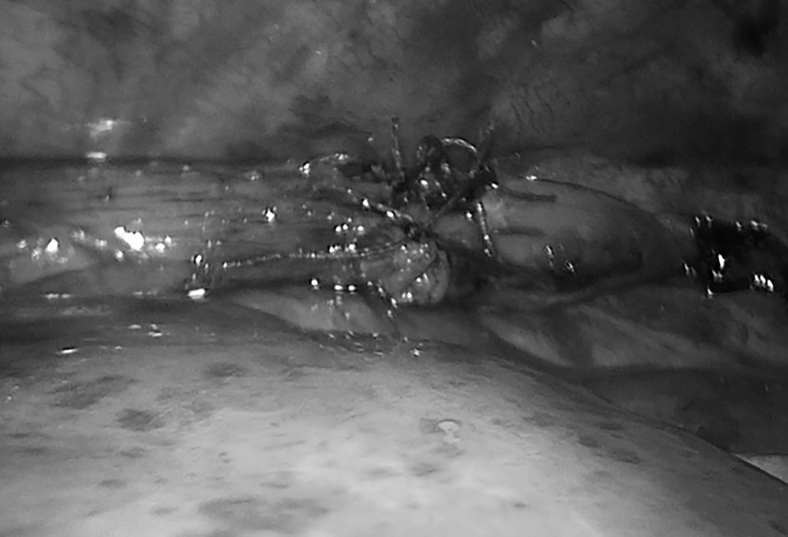
Esophageal anastomosis.

Clip-bearing fragments were resected and removed from the pleural cavity. The silicone 8F nasogastric tube was passed through the anastomosis. Esophago-esophagoanastomosis was made using absorbable sutures with a sliding suture technique (Fig. 3). The pleural cavity was drained. After surgery, the children were also sedated and myorelaxed; on the fifth postoperative day, the infants underwent an X-ray contrast examination of the esophagus, based on which the pleural drainage was removed and sedation was canceled and feeding was started. The study was approved by Nazarbayev University ethics committee.

## Results

Native esophagus preservation was achieved in all children by thoracoscopic internal elongation of the esophagus. The results are presented in the tables. According to an X-ray contrast study of the esophagus, anastomosis leakage was not observed in any patient, and feeding through a gastrostomy was started. All 3 children were discharged for outpatient treatment after the restoration of oral feeding. The follow-up averaged 10–13 months. Two cases underwent dilatation twice, 1 child had no stenosis. All patients had mild-to-moderate gastroesophageal reflux and receive conservative therapy. At the moment, all children feed by mouth and have a good nutrition status.

## Discussion

To overcome the gap between esophageal segments, tractional methods such as Foker and Kimura procedures and others have been proposed. The method of Kimura and Soper, which was first published in 1992, involves multistage extrathoracic esophageal lengthening. It describes the advancement of a cervical esophagostomy along the anterior chest wall subcutaneously in several stages until the length is sufficient for an esophageal anastomosis.^5^

The technique of Foker et al., which was described in the 1990s, entails external tractional elongation of the esophagus before anastomosis. The author hypothesized that the esophagus would grow if stimulated by traction.^2^ With the development of endoscopic surgery, the Foker procedure became available thoracoscopically, and in 2007 Van der Zee et al. reported 10 children operated on by this method.^1^

Magnet anastomosis is an interesting treatment for LGEA. Up to 2018, 7 patients of LGEA treatment using this method have been published. The essence of the method is that magnets are placed in the lower and upper pouches of the esophagus, which are attracted to each other, the distance is overcome, and a sutureless esophago-esophagoanastomosis is created. The main advantage of this method is that it does not require surgical interventions on the chest. The disadvantage of the method is the high incidence of early esophageal stenosis.^6^

Thoracoscopic esophageal elongation using internal traction sutures was developed at the Department of Pediatric Surgery and Urology, Medical University of Wroclaw, Poland.^7^ This technique consists of direct internal traction of the blind ends of the esophagus followed by repeated thoracoscopic approximation of the esophageal ends until anastomosis is possible. In the internal traction method, in contrast to the already presented concepts, one or two traction sutures were used only to bring the blind ends of the esophagus closer to each other, without their fixation on the chest wall.^8^

In our three observations, the use of the method, which was already mentioned, allowed for saving the native esophagus. Bogusz et al. reported the use of the method without gastrostomy among newborns,^8^ however, considering the fact that this is our first experience, gastrostomies were implanted for all children. When the method was first introduced, the interval between tractions averaged 41 days,^7^ but as the method improved, the time periods between tractions became shorter, so in 2020, Kozlov et al. reported 8 patients with an interval of 5 to 12 days.^9^

It is possible that lengthening time between tractions is not so important, because in our clinical cases, intervals between tractions of 2–3 days were sufficient for elongation of the esophagus and anastomosis application. Patients receive conservative therapy for gastroesophageal reflux disease, probably requiring surgical correction in the future. Since the number of LGEA cases is low, even some of the larger centers have limited experience. That is why we believe that the experience of 3 cases is not enough to perform this type of operation on our own, so we are planning further training to acquire the necessary skills.

## Conclusion

Thoracoscopic internal traction of the esophagus in the treatment of LGEA can be used not only in the neonatal period but also at an older age. Furthermore, an interval between tractions of 2–3 days seems to be sufficient for esophago-esophagoanastomosis.
